# Fertility-enhancing effects of *Prunus amygdalas *oil on reproductive functions of male rats: A two-generation study

**DOI:** 10.22038/AJP.2024.24590

**Published:** 2025

**Authors:** Sadia Suri Kashif, Sadaf Naeem, Saira Saeed Khan, Aisha Razzaque

**Affiliations:** 1 Department of Pharmacology, Faculty of Pharmacy and Pharmaceutical Sciences, University of Karachi, Karachi, Pakistan; 2 Faculty of Pharmacy, Ziauddin University, Karachi, Pakistan; 3 Institute of Pharmaceutical Sciences, Jinnah Sindh Medical University, Karachi, Pakistan; 4 Obstetrics and Gynaecology, Queen Medical Centre, Nottingham University Hospitals, Nottingham, United Kingdom

**Keywords:** Fertility, Sex hormones, Oxidative stress, Almond, Infertility models, Two-generation study, Ethanol

## Abstract

**Objective:**

*Prunus amygdalas *(Almond; PA) has extensively been used in traditional medicine and has been the source of robust phenolic compounds. The current study intended to evaluate the fertility-enhancing effect of PA on male rats infertility and reproductive performance of two successive generations of rats namely, F_0 _and F_1_.

**Materials and Methods:**

Chemical composition of the oil was established with the aid of Gas Chromatography-Mass Spectrometry (GC-MS). The oil was then subjected to *in-vitro* antioxidant assay via DPPH (1,1-diphenyl-2-picrylhydrazyl) and ROS (reactive oxygen species), followed by *in vivo* toxicity testing. In the fertility assessment, 1 and 2 ml/kg of PA oil was given to rats up to pre-cohabitation, cohabitation, gestation and lactation period. The reproductive performance along with hormonal and antioxidant markers of F_1_ generation was estimated and histopathological evaluation of both sex organs was done. Further, ethanol-induced male infertility model was established and sex hormones, antioxidant markers (superoxide dismutase (SOD), Glutathione peroxidase (GPx) and lipid profile were assessed along with histopathology of male sex organs.

**Results:**

The PA oil supplementation showed pronounced fertility outcomes in terms of raised litter size, sex hormones and antioxidant markers in both generations. Moreover, in ethanol-induced male infertility model, PA oil significantly restored sex hormones, ROS and GPx levels. Histological findings also endorsed better spermatogenesis with enhanced architecture.

**Conclusion:**

These results strongly suggest that PA oil rich in PUFA (poly-unsaturated fatty acids) might be a promising treatment option in future for male/female sterility.

## Introduction

Fertility is dependent on environmental, genetic, hormonal and lifestyle factors. Approximately 10 to 15% of couples are impacted by infertility. Infertility is one of the growing health problems worldwide among couples. About 35% fertility cases have been attributed to male factors, such as erectile dysfunction and sperm motility (Ramgir et al., 2022). Recent studies nowadays correlates male infertility with dietary habits, lifestyle changes, lack of physical activity and weight gain. Male infertility arises owing to sperm delivery problems associated with reduced sperm production, tract blockages or abnormal functions of reproductive organs (Mousavi et al., 2021). It appears that diet rich in PUFA, especially omega-3 and 6, determined an increase in mitochondrial energetic metabolism and a reduction in oxidative damage that improve sperm quality (Ferramosca and Zara, 2022).

Oxidative stress, produced from excess free radical and ROS (reactive oxygen species) production, causes complete or partial dysfunction of male reproductive system (De Luca et al., 2021). ROS generation causes infertility by damaging the sperm membrane which in turn reduces the sperm's motility and ability to fuse with the oocyte. In addition, ROS also impair fertilization via altering sperm antioxidant defense system leading to sperm DNA damage (Tremellen, 2008). Adding antioxidants along with hormonal treatment during preparation techniques to the sperm medium is a treatment strategy to help treat infertility (Majzoub and Agarwal, 2017). Different natural antioxidants have expressed promising effects against male infertility especially ginseng, cinnamomum, rose oil, ginger, onion, sesame, flex seeds, walnut, etc. (Akbaribazm et al., 2021). Among them, nuts are rich source of poly unsaturated fatty acids. Poly unsaturated fatty acids (PUFAs) such as omega 3, omega 6, eicosapentaenoic acid (EPA) and docosahexaenoic acid (DHA) are natural antioxidants particularly important for sperm motility, normal morphology, plasma membrane integrity and freezing resistance (Yuan et al., 2023). PUFAs as phospholipids are essential component of spermatozoan membrane, and they improve semen quality and sperm susceptibility to lipid peroxidation (Rodak and Kratz, 2023). It was also reported that fish oil supplementation improved sperm motility and viability parameters in male rabbits (Collodel et al., 2020; Gliozzi et al., 2009). Similarly, Khani et al*.* reported that sesame seeds as a rich source of PUFAs improved sperm count and motility, and can be effective for male infertility treatment (Khani et al., 2013).

The nuts, fish and vegetables are enriched with significant amounts with PUFAs (Pollicino et al., 2023). Nuts, including almonds, are highly enriched with PUFAs, MUFAs (Monounsaturated fatty acid) and ALA (α-Linolenic acid), which are responsible to regulate follicular fatty acid concentrations and fatty acid concentrations in sperm membrane in females and males, respectively. Moreover, nuts enhance antioxidant capacity by potentiating anti-inflammatory pathways. During spermatogenesis, sperm cells in seminal fluid are extremely sensitive to oxidative stress, thus antioxidants decrease sperm DNA fragmentation (Cardoso et al., 2023). 

PUFAs have a huge impact on animal reproduction including the regulation of steroidogenesis, prostaglandins synthesis, membrane properties and transcription factors. It has been reported that PUFAs sources supplementation can positively influence sperm characteristics that enhance male fertility. Steroidogenesis and spermatogenesis of the testis depend on various gonadal hormones. PUFAs and metabolites of PUFAs take part in steroidogenesis by affecting steroidogenic acute regulatory protein (StAR) and cholesterol cleavage enzyme (P450scc), responsible for maintaining steroid synthesis. StAR encourages cholesterol movement from external to the internal mitochondrial membrane, while P450scc causes cholesterol to pregnanolone conversion to initiate steroidogenesis (Qi et al., 2019). PUFAs are vulnerable to lipid peroxidation that can induce ROS production, resulting in sperm damage leading to reduction in sperm functioning (Zanussi et al., 2019). 

Almond (*Prunus amygdalas*; PA) contains high concentration of PUFAs as well as monounsaturated fatty acids (MUFAs). They are naturally highly loaded with riboflavin, vitamin E, calcium, zinc, phosphorus, potassium, manganese and copper (Richardson et al., 2009). It was reported to improve fecundity because all these chemical compositions lead to hypocholesteremia and reduced risk of diabetes and oxidative stress. Almonds possess an antioxidant role as it contains zinc needed for sperm motility, acrosomal exocytosis, capacitation and maturation. In females, zinc is required in processes that regulate oocyte maturation, follicle development and fertilization. Moreover, zinc deficiency decreases serum testosterone concentration leading to cessation of spermatogenesis (Chao et al., 2023). Testosterone is a critical regulator in the differentiation and development of secondary sexual characteristics and male sex organs. Follicle-stimulating hormone (FSH), luteinizing hormone (LH) and Gonadotropin hormone-releasing hormone (GnRH) regulate testosterone synthesis in Leydig cells of testis (Cardoso et al., 2023). Hence, it may be postulated that *Prunus amygdalas* can enhance fertility.

Traditional medicines and herbal remedies are widely used in developing countries as they are affordable and have easy access and fewer adverse effects than chemical drugs. Researchers nowadays are focused on naturally existing compounds as they are healthier and have fewer adverse effects (Rad et al., 2021). For this purpose, recently, we explored fertility enhancing role of *Juglans regia* nut oil in a bi generational study that confirms PUFAs-containing oil possesses fertility-enhancing properties (Suri et al., 2023). In continuation of this exploration, the current study was designed to assess the protective and beneficial use of* P. amygdalas *oil on male fertility and sex hormones in experimental animals.

## Materials and Methods

The almonds (*Prunus amygdalas*) were purchased from Quetta, Pakistan, and identified at the University of Karachi, Herbarium and Botanical Garden, Department of Botany (GH 95589). Fruits were then cracked and shelled manually, and exposed to expeller pressing to obtain oil. This process of oil extraction, without any chemical, is the oldest and simplest method and the pure oil is extracted with little operation cost.

### Drugs and chemicals

Drugs and chemicals used were of analytical grade. Ferric Chloride, Petroleum ether, Ethanol (96%), 10 % Formalin, Potassium hydroxide, Glacial acetic acid, Sulfuric acid (concentrated), Sodium Hydroxide, Mayer’s and Dragendorff’s reagents, HCl 1%, Cholesterol reagent, Conjugate solutions for testosterone and estradiol, Tetramethylbenzidine (TMB), Stop solution, HRP (Horseradish peroxidase) Conjugate reagent, FBS (Fetal bovine serum), PBS (Phosphate-buffered saline), Na2CO3 (30 g/L) were procured from Sigma Chemicals Company (St Louis, MO, USA). Vitamin E 105 IU/day (DL-α-Tocopherol acetate), Ascorbic acid, dimethyl benzene, Ketamine/ Xylazine, Etoposide, 0.1 mM DPPH, DMEM (Dulbecco’s Modified Eagle Medium) and 1% Penicillin-streptomycin, were procured from MERCK and Sigma–Aldrich Co., USA. Gallic acid and Folin-Ciocalteu reagent were obtained from Merck (Darmstadt, Germany). Olive oil was purchased from Saeed Ghani Ltd., Pakistan. All chemicals used in this study were of analytic grades. 

### Determination of total phenols

Determination of total phenols in oil was carried out by utilizing Folin-Ciocalteu method as previously described (Neo et al., 2010). Gallic acid (GA) was used as a standard. The results are expressed as gallic acid equivalents per gram (mg/g) (Yanai et al., 2018).

### Preliminary phytochemical testing

PA oil were subjected to qualitative phytochemical screening using previously described standard procedures. The methods employed with few modifications in accordance with the previous studies (Harborne, 1998; Jaradat et al., 2015).

### GC-MS analysis

The GC-MS analysis was performed with reference to a published method (Al-Owaisi et al., 2014). GCMS-QP2020 NX system (Shimadzu Co., Japan) along with autosampler was used. A sample solution of 0.3 μL was inserted to Rtx-5MS column (30 m×0.25 mm×0.25 μm). Helium was used at a constant flow rate (1.0 mL/min) as a carrier gas. The temperature of ion source, injection and transfer line were maintained at 250ºC. The mass spectra were obtained from 40 m/z to 1000 m/z. The column temperature initially was held at 40ºC. After 15 min, the temperature was increased at 3ºC/min up to 300ºC. The spectra were matched with standards available in spectrum libraries.

### ROS analysis

The DCF-DA (2'-7'-Dichlorodihydro fluorescein diacetate (DCFH-DA) method was used to assess % Viability and ROS production of PA oil on primary cortical neuronal cells. Primary cortical cells were used for the determination of Reactive Oxygen Species generation. The method was adopted with some modifications (Leiros et al., 2014; Suri et al., 2024; Tarasenko et al., 2012). The untreated cells were used as a control. Cortical cells were harvested from the brain of two-day-old rat pups. Cells were grown in DMEM affixed with 10% FBS and then 1% penicillin-streptomycin (100 units/mL-100 μg/mL) respectively. Adhesive cell monolayers were cultured into T25 tissue culture flasks to be used for further experiments. The cortical cells were incubated at 37˚C in a humidified atmosphere having 5% carbon dioxide (CO2). ROS intracellular levels of living cells were assessed quantitatively by employing a fluorescent probe, 20,70 dichlorodihydrofluorescein diacetate (DCF-DA). Primary cortical cells were subjected to various concentrations of sample for 12 h. Untreated or treated cultures were allowed to incubate in the dark with 10 μM DCF-DA at 37˚C for 45 minutes, and later on, washed with PBS. Relevant ROS level changes intracellularly were observed by fluorometric detection of DCF by using a fluorescent Microplate Readers RE, ver. 5.0.0.42 at emission and excitation wavelengths of 530 nm and 485 nm, respectively. The fluorescence intensity level of DCF is proportional to the ROS level generated intracellularly. Percentage Cell Viability can be calculated by formula (Kamiloglu et al., 2020).

### DPPH analysis

An upgraded DPPH assay (1,1-diphenyl-2-picrylhydrazyl) was selected to evaluate the tested compound. The DPPH radicals diminish at 517 nm of absorbance in the presence of antioxidant potential. The test compound at different concentrations (10, 20, 100, 300 μg/ml) was added to 0.1 mM DPPH (3 ml) and allowed to react. The mixture was swirled and incubated for 30 min at room temperature. A microplate reader (SkanIt Software 5.0 of RE, ver. 5.0.0.42) was utilized to measure the absorbance at 517 nm. The test compound reducing power was calculated with the absorbance of ascorbic acid, which served as control. (Alshammari et al., 2022).

Radical Scavenging Activity was calculated via the following formula.

% Radical Scavenging Activity (RSA) = [(Ab_control_ -Ab_sample_) / Ab_control_] × 100

### Brine shrimp toxicity analysis

The safety of the oil was monitored by the brine shrimp lethality test to predict the presence of cytotoxic activity. The assay system was conducted by preparing 10 bijoux bottles filled with 2 mL of seawater each and a two-fold dilution was set up to yield a series of concentrations from 100 to 0.195 mg/ml. Potassium dichromate was dissolved in artificial seawater and functioned as a positive control with concentrations ranging from 0.1 to 0.9 mg/ml. An aliquot (0.1 ml) containing about 10 to 15 Artemia salina nauplii was introduced to each bottle and observed for 24 h. The dead larvae from each bottle were counted after 6 and 24 h. After 24 h, numbers of survivors were counted and data were used for LD_50_ determination as a standard drug Etoposide (Arulvasu et al., 2014; Meyer et al., 1982).

### Acute toxicity

Acute oral toxicity of PA oil was assessed with reference to the OECD guidelines (OECD, 2001). Both genders were utilized and were provided with distilled water and feed *ad libitum*. Twenty healthy and mature rats were grouped randomly in four groups (n=5) comprising of a control group and three treated groups. *Prunus amygdalas* oil was administered in a single oral dose of 100, 1000 and 2000 mg/kg bw to different groups, and compared to control group. The rats were inspected for mortality and toxicity signs if any, like behavioral changes for 48 h (Hazarika et al., 2019; Lalitha et al., 2012).

LD_50 _of *Prunus amygdalas* can be calculated as:

LD_50_ = Highest Dose – Σ (Mean Mortality × Dose Difference) /n 

LD_50_ = 2000 – (0 + 0 + 500) / 5 

LD_50_ of *Prunus amygdalas* Oil = 1900 mg/kg/BW

### Ethical approval

Experimentation was executed after approval by the Board for Advanced Studies and Research, University of Karachi and Pharmacology Departmental Research Committee (reference No 05071/pharm) according to the National Institute of Health guidelines, Pakistan (Council, 2010; Garber et al., 2011). 

### Bi-generational study

Animals

Male and female adult Wistar rats weight 160-180g were used for fertility assessment. The animals were purchased from the H.E.J animal house, University of Karachi, having appropriate ventilation with standard temperature (25 to 30^o^C) and 12-hr dark/light cycle. 12 weeks old male rats and 14 week old female rats were used for the study and allowed for acclimatization period of seven days. Animals were paired and housed in polypropylene cages (29 × 18 × 13 cm) with wood shavings as bedding. The experimental protocol fulfills the guidelines on the appropriate use and care of laboratory animals (Council, 2010). 

### Experimental protocol

The study conducted for a period of seven months in reference to a modified method (Vohra and Khera, 2016). Eighteen pairs of rats (F_0_ generation; n=36) were placed in separate cages.

In the fertility study, sexually mature rats were used. Rats were isolated and evaluated for any gross symptoms of injury or disease. After one week of acclimatization, six experimental groups were formed. Three groups were of male rats, each comprising of six rats (n=6), while the rest of the three groups comprised of female rats only (n=6).

In male rat groups, Group I was administered distilled water daily, and served as control, while, Group II animals were treated with1 ml/kg/day of PA oil, and Group III animals were given PA oil, 2 ml/kg/day (Hussein and Raheem, 2015). A similar protocol was followed for the female group. All rats were dosed for 30 days before mating (Pre- Cohabitation) by gastric gavage between 14:00 and 16:00 hr. During mating, each male rat was individually paired with female rats of each group in separate cages for seven days (1:1). Inseminated females were separated from males and placed in cages after conception. The doses of PA oil were given continuously to female rats during cohabitation (21 days), during gestation (21 days), delivery and during lactation till postnatal day of 22. Gestated female rats were permitted to naturally deliver pups (F_1 _generation). Fertility indices (number of pups, survival index, fertility index) were estimated. Offspring were observed for any abnormality on post-delivery days. All male/female F_0_-parental animals were twice daily monitored for toxicity signs.

For F_1_ generation study, pups were grown up to 4 weeks then the same doses of the PA oil were given to 4 week old pups (according to their body weight) during their growth up to breeding. After one month, 8 week old rats were permitted mating by making pairs on a 1:1 basis within same treatment group, preventing sibling-mating, for 7 consecutive days. The presence of vaginal plug confirmed the pregnancy in rats. Pregnant female rats of the F_1_ generation were separated from male rats and placed individually. PA oil was continuously given during all phases including pre-cohabitation, cohabitation, gestation up to lactation, until the F_2_ generation offsprings were weaned. Gestated female rats were permitted to naturally deliver pups (F_2 _generation).

Afterward, when offsprings were delivered, all F_1_ animals (male and female) were deeply anesthetized with intraperitoneal ketamine (10 mg/kg) and xylazine (5 mg/kg) (Joksimovic Jovic et al., 2021), to alleviate the suffering of rats. Blood samples were taken from the aorta and the animals were sent to necropsy. Subsequently, reproductive organs were reserved for histopathological assessment.

 The same protocol of the F_0_ generation was followed for both F_1_ and F_2_ generation till the birth of F_2_ pups. For all F_1_ generation female rats' fecundity assessment, the above procedure for determining fertility indices (in F_0_ generation animals) were used as described by Vohra and Khera (Vohra and Khera, 2016). 

### Reproductive indices of rats

The reproductive indices of female rats were studied and analyzed at all stages initializing from conception till weaning of pups and computed by modified methods (Narayana et al., 2005; Vohra and Khera, 2016; Walker and Cheng, 2005).

### Blood parameter assessment

Samples were centrifuged for 10 min at 4000 rpm. The serum was stored at -80^o^C for biochemical and hormonal testing. All samples were assayed via ELISA Roche Diagnostics, Basil (Alam, 2019). Glutathione and SOD were assayed by the kit obtained from Glory Science Co., Ltd. (Glory Science Co.2022#45).

For histopathological evaluation, the ovaries and testis of the selected male and female rats were removed, fixed in 10% formalin and processed (Tassinari et al., 2021).

### Ethanol-induced male model of infertility

The experimental fertility study used male Wistar rats (160 to 200 g) kept in proper ventilation cages with a 12 hr light/dark cycle and housed at 25 to 30^o^C.

For male infertility model establishment, modified method was employed (Oremosu and Akang, 2015). The animals were placed randomly in four different groups (n=6); normal control, negative control group (2 g/kg/bw Ethanol), 2 ml/kg/day PA oil group (Hussein and Raheem, 2015) and positive control Vitamin E group at a dose of 105 IU daily (Leskovec et al., 2019). All rats were dosed by gastric gavage tube. All groups were dosed with 2 g/kg ethanol daily for up to 8 weeks excluding control group.

Afterward, all animals were deeply anesthetized as described earlier and blood samples were taken and placed under aseptic conditions. The samples were centrifuged in an Eppendorf machine at 4000 rpm for 10 min. The separated serum was stored at -80^o^C. All blood samples were analyzed for SOD and glutathione (Glory Science Co.,2022#45), Total lipid profile, hematological parameters via Sysmex KX-21 hematological analyzer (Fares, 2001), hormonal estimation via ELISA Roche Diagnostics, Basil (Sultana et al., 2020). 

For all male rats' fecundity assessment, the procedure described by Quadri and Yakubu (Quadri and Yakubu, 2017) was selected to prepare the semen from the epididymis. For histopathological evaluation, the testes were removed, cleared from adherent tissues and processed as described previously. The stained slides were examined and compared among the groups (Tassinari et al., 2021).

### Statistical analysis

Statistical analysis was conducted by SPSS version 20 software. Data was analyzed by one-way ANOVA and Bonferroni *post-hoc* test. Values were considered to be statistically significant at the level of p<0.05.

## Results

### % Yield and total phenol of PA oil

The percentage yield PA oil was 45.37 % from 8 kg of fruits. Total Phenolic Content (TPC) was determined by the Folin–Ciocalteu (F–C) method, TPC expressed in terms of mg/g GA that was found to be 0.03 mg/g of GA and 3.12% (w/w) respectively (Table 1). The total phenolic contents were calculated using the linear equation based on the calibration curve of gallic acid.

### Preliminary phytochemical screening

Preliminary phytochemical analysis of the oil showed the presence of major phytochemicals like glycosides, phlobatannins, flavonoids, terpenoids, etc. (Table 2). Saponins, alkaloids and amino acids were not detected in the PA oil. 

### GC-MS analysis

**Figure 1:**
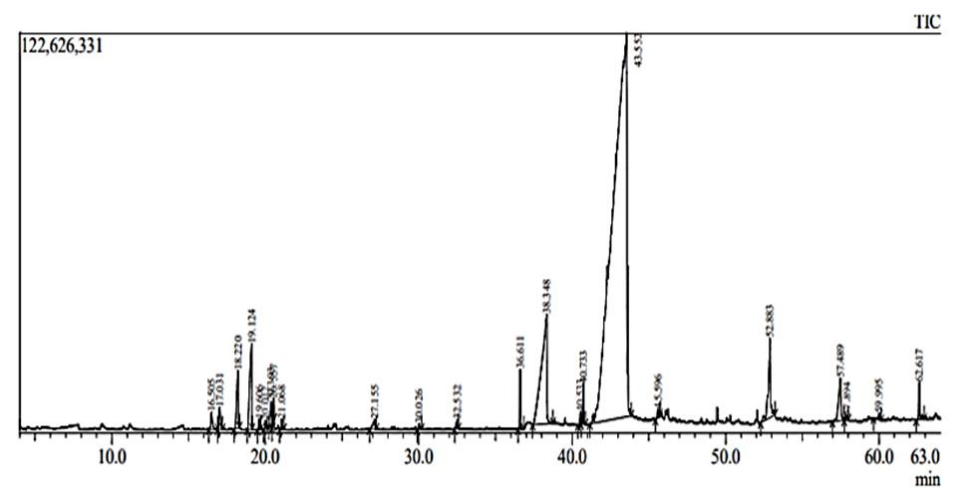
GC-MS complete chromatogram of *Prunus amygdalas* oil

**Figure 2:**
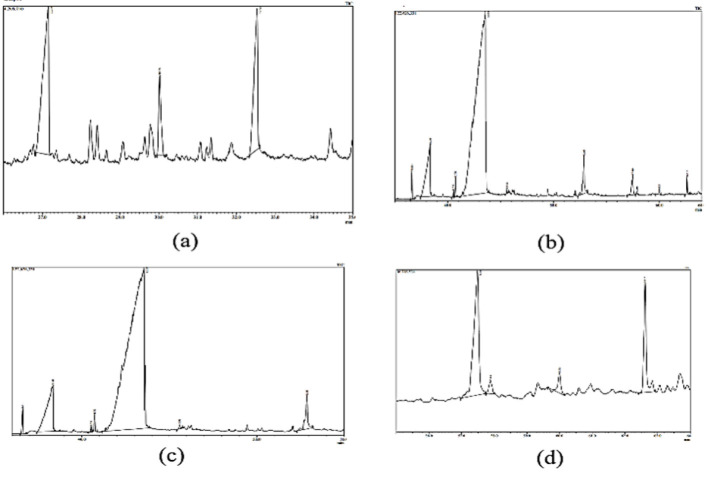
(a) Chromatogram peaks at 26-35 min, (b) Chromatogram peaks at 35-64min, (c) Chromatogram peaks at 36-55 min, (d) Chromatogram peaks at 55-64 min

GC-MS analysis of PA oil displayed the presence of many polyunsaturated, saturated fatty acids and volatile compounds (Table 3). Figure 1 shows complete chromatogram of PA oil while Figure 2 shows chromatogram peaks at various intervals. The most abundant fatty acids in the almond oil were oleic; α-linoleic, stearic, linolenic and palmitic acids respectively. Other fatty acids were of trace quantities. The oils contained mostly monounsaturated acids followed by saturated fatty acids. Further volatile compounds like nonen, octanal, undecane, nonanal, dodecane, etc. were also identified by GCMS analysis.

### Antioxidant assays

ROS 

**Figure 3:**
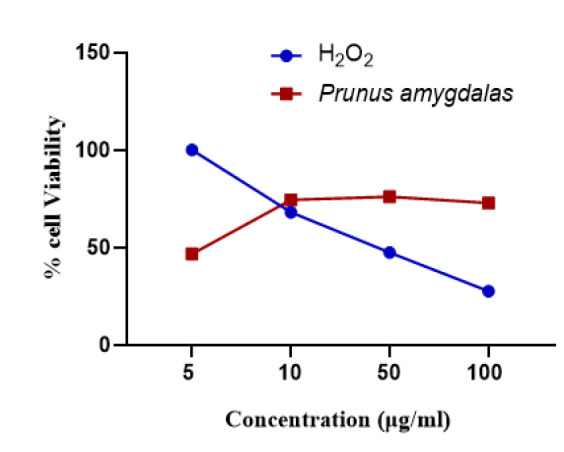
Percentage Viability of *Prunus amygdalas *in comparison to H_2_O_2_

The method revealed that it was nontoxic and prevented H_2_O_2_-induced oxidative damage significantly. % Cell viability after incubation using indicated concentrations of *Prunus amygdalas *oil is mentioned in Figure 3, that showed its protective efficacy against H_2_O_2_-induced neuronal toxicity and no cytotoxic effect was found up to a dose of 100 (µg/ml) in neuronal cells. 

### DPPH

The scavenging effect of different concentrations of PA oil on the DPPH free radical was compared with standard antioxidant, ascorbic acid. The results are expressed as inhibition (%) in Table 4. Oil showed a dose-dependent scavenging activity at different concentrations as highest inhibition was seen at 10 μg/ml (58.10±2.85%) while ascorbic acid exhibited the strongest scavenging activity 88.24±1.29% at 300 μg/ml.

### Brine shrimp testing

The results of brine shrimp lethality test are shown in Table 5 and showed that PA oil is safe at various concentrations. No significant toxicity to brine shrimp (*Artemia salina*) was found. The % mortality of *P. amygdalas* at 1000 μg/ml concentration was 6.66 % compared to standard Etoposide.

### Acute oral toxicity

Acute toxicity testing showed that PA oil is safe even at higher doses, LD_50_ values for oral administration of PA oil was found to be 1900 mg/kg/day.

### Bi-generational study

Breeding rate

F_1 _and F_2_ bi-generational study parameters are summarized in Table 6. In each generation, we did not find congenital abnormality in any group. One-way ANOVA revealed that the number of rat pups/litter was insignificantly increased in all treated groups of F_1_ generation (p>0.05). However significantly increased results in comparison to control group were seen in F_2 _generation rats (df 5,30; F 6.61; p<0.05).

### Reproductive indices of rats

Table 7 presents the reproductive indices in treated groups’ that showed live birth, fertility, and survival indexes found to be insignificant as compared to control group (p>0.05). While, a significant enhanced effect in litter size was observed in both generations’ animals (p<0.05) when compared to control group.

### Blood parameter assessments

**Hormonal assessment**

**Figure 4:**
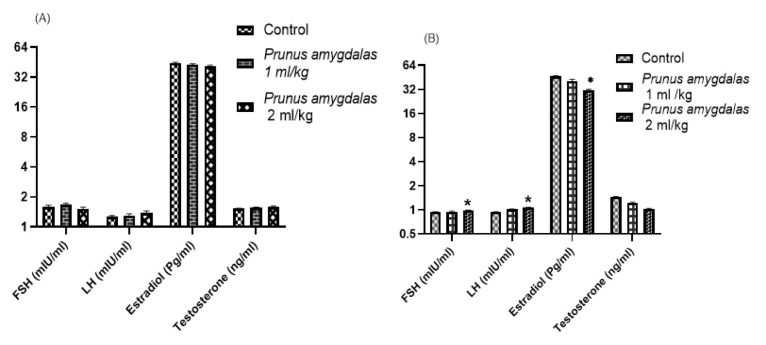
Effect of *Prunus amygdalas *on Hormonal Parameters as compared to control: Male (A); Female (B). n=6, Mean±SEM; *p<0.05 significant, as compared to control.

Figure 4 presents the effect of low and high doses of PA oil on hormonal parameters in rats. The results of this study showed that the administration of PA oil significantly increased LH and FSH levels in female group compared with control and male group (p>0.05). In contrast, insignificant changes were seen in male rats with respect to LH, FSH, testosterone and estradiol levels. Additionally, testosterone levels were insignificantly raised in male rats in comparison to control group. 

### GPx and SOD assessment

**Figure 5:**
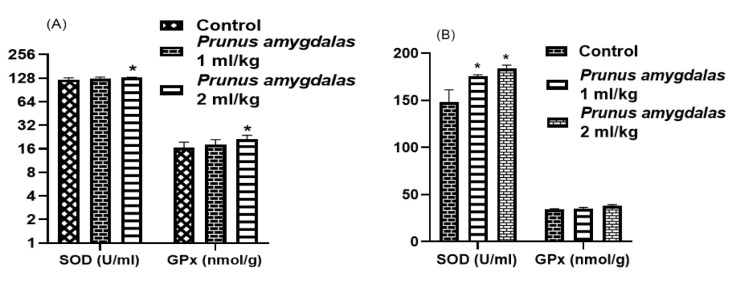
Effect of *Prunus amygdalas *on Oxidative Parameters as compared to control: Male (A); Female (B). n=6, Mean±SEM; *p<0.05 significant, as compared to control.

Figure 5 presents the effect of PA oil on oxidative parameters. The results of this study showed that the administration of PA oil significantly increased SOD levels in male rats at high dose (p<0.05) in comparison to low dose and control group. While female rats showed significant SOD levels (p<0.05) at both doses as compared to control group. In contrast, GPx levels were found to be raised in male rats (p<0.05) in comparison to female and control rats. 

### Histological evaluation

**Figure 6:**
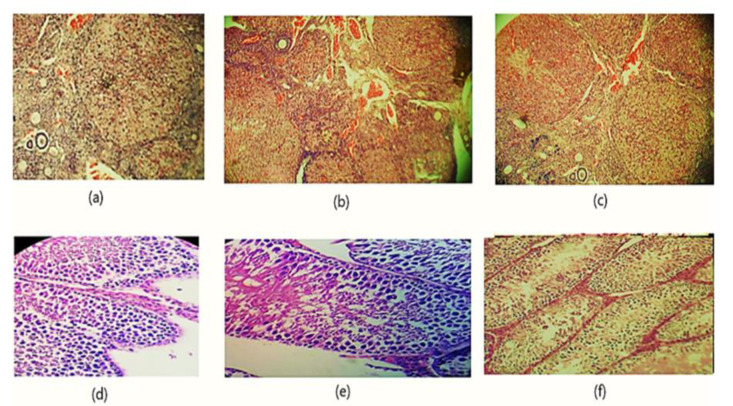
Effect of *Prunus amygdalas* on Histopathological Parameters (a) Micrograph rat ovary of control (10x), (b) Micrograph rat ovary low dose *Prunus amygdalas* treated (10x), (c) Micrograph rat ovary high-dose *P. amygdalas* treated (10x), (d) Micrograph rat testis of control (10x), (e) Micrograph rat testis low-dose *P. amygdalas* treated (10x), (f) Micrograph rat testis high dose *P. amygdalas* treated (10x)

The effect of PA oil on histological parameters is displayed in Figure 6. (a) Control animal ovaries showed normal architecture with profuse stroma and connective tissues in medulla. (b) low-dose PA oil treated ovaries were similar to control animal ovaries (c) high-dose PA oil treatment revealed the presence of Graafian follicle, antral follicles and corpus luteum. (d) Control group testis revealed occasional sertoli and spermatogenic cells with tiny connective tissues at surroundings. (e) low-dose oil treated group displayed normal seminiferous tubules like control group. Correspondingly, (f) testis treated with high dose PA oil, displayed normal architecture of seminiferous epithelia.

### Ethanol-induced male infertility model

**Figure 7:**
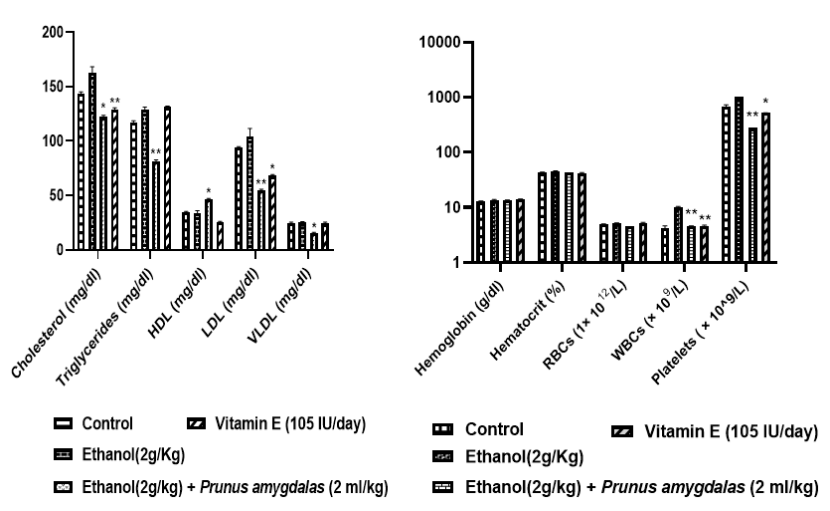
Effect of *Prunus amygdalas *on Biochemical and Hematological Parameters. n=6, Mean±SEM; *p<0.05 significant, **p<0.01 highly significant, as compared to negative control.

Table 8 presents the parameters like semen pH, sperm count, sperm motility, sperm viability, and semen volume. All these parameters were significantly improved after PA oil low and high dosing for 60 days (p<0.05) in all treated male rats as compared to the negative control group. The effect of the PA Oil on hematological and biochemical parameters for 60-day dosing is summarized in Figure 7. On biochemical parameters, PA oil produced a highly significant effect by decreasing triglycerides (TGs) and low-density lipoprotein (LDL) (p<0.01), as compared to the negative control group. 

Additionally, Very-low-density lipoprotein (VLDL) was significantly decreased (p<0.05) in comparison to the negative control group. Moreover, high density lipoprotein (HDL) levels were significantly increased (p<0.05) in comparison to the negative control group. 

Further PA oil consumption in rats showed a significant declined effect on white blood cells (WBCs) and platelets as compared to the negative control group. Moreover, insignificant results were obtained on levels of hemoglobin, hematocrit and red blood cells (RBCs) (p>0.05).

**Figure 8:**
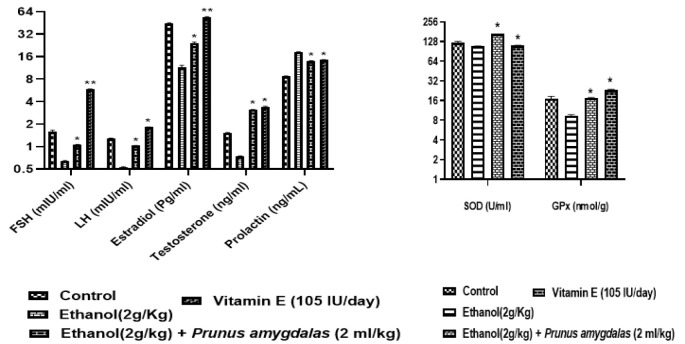
Effect of *Prunus amygdalas *on Hormonal and Oxidative Parameters. n=6, Mean±SEM; *p<0.05 and **p<0.01 significantly different from negative control group.

The effect of PA oil on sex hormones and oxidative parameters in male rats after 60-days dosing is summarized in Figure 8. PA oil administration exhibited a significant increase on FSH, LH, estradiol and prolactin levels as compared to the negative control group (p<0.05). The standard vitamin E showed similar significant increased results on FSH and estradiol, as compared to negative control group (p<0.01).

Oxidative parameters SOD and glutathione significantly improved in PA oil-treated animals (p<0.05), similar to standard Vitamin E, as compared to negative control group.

### Effect of PA oil on histopathological parameters

**Figure 9:**
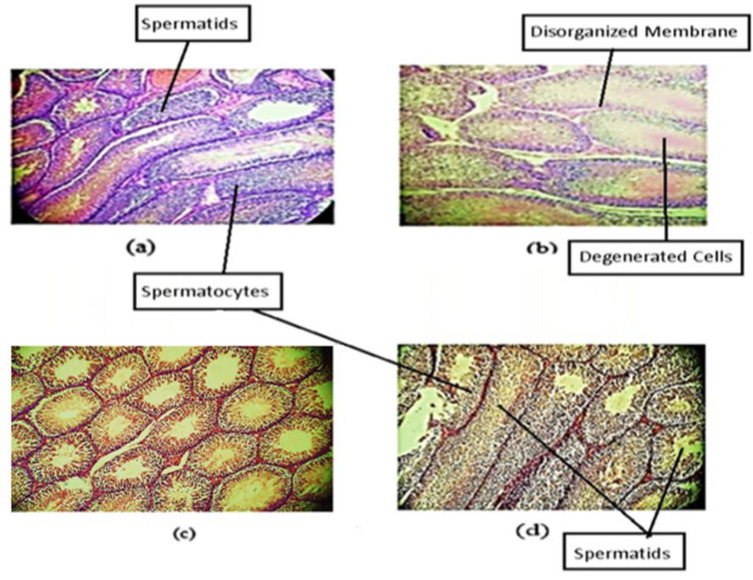
Effect of *Prunus amygdalas* on Histopathological Parameters. (a) Micrograph rat testis of control (20x), (b) Micrograph rat testis Ethanol treated (20x), (c) Micrograph rat testis treated with Ethanol and *Prunus amygdalas* (20x), (d) Micrograph rat testis treated with Ethanol and Vitamin E (20x)

Figure 9 shows the effect of PA oil on histopathological parameters of rat’s testis. Microscopic examination of the control group testis figure (a) showed tubular seminiferous epithelia normally present with occasional Sertoli cells. Normal architecture of the testicular tubules with various forms of germinal cells having filled lumen with spermatozoa. While the negative control group exhibited deteriorated germinal cells, detachment and extensive deterioration of seminiferous tubules which displays disorganized and irregular membranes. Histopathology sections of PA oil treated group testis, showed partial damage of germinal cells with normal appearance of spermatogenic epithelium, that presents a consistent arrangement of spermatogenic cells when compared to ethanol-treated animals. Vitamin E treated sections of testis also exhibited normal seminiferous tubules with occasional Sertoli cells similar to the control group. Closer to the central part of tubule, primary as well as secondary spermatids were observed. PA oil treatment showed enhanced production of spermatozoa and spermatids as compared to the negative control group.

## Discussion

Plant-based medicines have been men’s ultimate therapeutic option since ancient time and is still in the frontline for improving human health (Bykowska-Derda et al., 2023). PA has been used as antidepressant, antioxidant, memory enhancer and lipid-lowering agent (Morvaridzadeh et al., 2022). In the present study, we evaluated the fertility potential of the PA nut oil in two generational study and ethanol induced male infertility model. Preliminary phytochemical analysis represented the occurrence of flavonoids, glycosides, phlobatannins, terpenoids and phenols. Moreover, the quantitative data represents the presence of 0.03 mg/g GAE total polyphenolic content. Chen et al. reported almond is a rich source of poly unsaturated fatty acid, zinc, potassium and proteins (Chen et al., 2005). Phenolic acids, lignans, glycones, tocopherols, isoflavones, catechins and stilbenes are abundantly present in almond and its skin (Barreca et al., 2020). Presence of these compounds suggests antioxidant potential of almond oil. Further GC-MS analysis represented the presence of several PUFAs especially linolic acid, stearic acid, palmitic acid, octadecanoic acid, hexadecenoic acid, alpha tocopherol etc. Additionally, we have found presence of volatile compounds like nonen, octanal, undecane, nonanal, dodecane, etc. (Table 1, Figure 1, 2). Current results are in accordance with Qureshi et al. who identified volatile constituents and several PUFAs through GC-MS analysis of the hexane and chloroform fractions of almond (Qureshi et al., 2019). Moreover, Esonye et al*.* found PUFAs, MUFAs and volatile compounds in almond oil extracted through cold press (Esonye et al., 2019). 

Antioxidant potential was determined by percentage viability testing via ROS production and DPPH testing. Results indicate profound non-toxic, antioxidant role of PA oil in comparison to H_2_O_2 _as we found improved neuronal cell viability as shown in Figure 3. The antioxidant activity of PUFAs containing food may be related to their redox properties which allow them to act as reducing agents or hydrogen-atom donors, or their ability to scavenge free radicals (Esfahlan and Jamei, 2012). Similarly, in DPPH test, PA oil showed 58.10% inhibition that showed its highest free radical scavenging activity at lowest concentration. Antioxidants present in natural compounds scavenge ROS, may be of great value in preventing the onset of oxidative stress and its related diseases (Sarwar et al., 2012).

Furthermore, brine shrimp lethality testing showed that PA oil is safe at low concentration (6.6 % lethality) and even at higher concentrations. Study suggested that PA has useful potent bioactive, non-toxic compounds that can be useful as therapeutic drugs in different ailments. Likewise, in-vivo acute toxicity testing showed PA oil exhibited LD_50_ values up to 1900 mg/kg/bw and no change in body weight, behavior and appearance were observed during 48 hours up to 2000 mg/kg/bw dose. These results are in accordance with previous researches that have been defined as nuts especially almond as an edible food demonstrates the non-toxic nature even at higher doses that encouraging the further exploration of its potential for safe consumption and its health benefits. (Lebaratoux et al., 2016; Oseh et al., 2019).

PA oil is a rich source of PUFAs, and researches have reported that omega-6 and omega-3 PUFAs are essential component of reproductive cycle, including oocyte maturation, spermatogenesis, sperm motility and embryo implantation (Lass and Belluzzi, 2019). In the two generational study, numbers of pups production were increased after 7 month dosing in both F_1_ and F_2_ generations. These findings are in agreement with Rehman et al*.* who reported that PA has beneficial effects on male fertility as its oil efficiently improved sperm morphology and testosterone levels against atenolol induced infertility model (Rehman et al., 2021; Rehman et al., 2022). Moreover, Shabbir et al. reported that PA oil is beneficial for female infertility as it possesses hypolipidemic, hypoglycemic and anti-androgenic effect that restored ovarian morphology in letrozole-treated rats (Shabbir et al., 2019). Current study observed increased litter size in F_2_ generation. However, slightly raised insignificant results were seen after 21 days on live birth index, viability index, mating indices and fertility indices. 

It is reported that PUFAs supplementation has influence on biosynthesis of prostaglandin and steroidogenesis that have multiple roles in the reproduction. LH and FSH are sex hormones that are required for both male and female fertility processes. Irregularities of FSH and LH hormone lead to immature follicles and egg production which results in infertility. Additionally raised LH levels produce abnormal testosterone, estrogen and androgen that results in infertility (Ferguson and Leese, 2006; Wang and Voronina, 2020). 

We have found significantly high levels of FSH and LH in female rats (Figure 4) while male rats showed insignificant changes in these hormones in comparison to control. These finding agreed with Jahromi et al, who reported fertility enhancing effect and significant sex hormones regulation with a polyherbal formulation (Loboob) that contained *Prunus amygdalas* along with other herbal ingredients (Jahromi et al., 2023).

It is well known that oxidative stress is a pivotal reason for the infertility as free radical formation, especially ROS, upsets the homeostatic balance between free radical formation and antioxidant capacity that disturbs sex hormones and produces infertility in humans (Takeshima et al., 2021). In the present study (Figure 5), significantly raised SOD levels were observed after both low and high doses of PA oil. Male animals presented significantly raised levels of glutathione. However, females exhibited same values as control in low doses, while high dose administration showed slightly insignificant raised amount of glutathione that validate the dose dependent anti-oxidant potential of PA*.* Our findings agreed with the report of Bouaziz et al. who reported that almond contains appreciable amounts of polyphenols and PUFAs that exert highest antioxidant activity in DPPH assay even higher to BHA standard (Bouaziz et al., 2017). This potential of PA oil confirms that oxidative stress-related infertility can be ameliorated by daily consumption of almond that thought to reduce the damage caused by oxidative stress.

Further PA oil’s potential against male infertility was evaluated by establishing ethanol-induced male infertility model. Testosterone is a male hormone that involves in spermatogenesis, it is secreted by the Leydig cells of testicles responsible for sperm production and maturation (Dohle et al., 2003). In the current study, ethanol successfully produced infertility in all male animals that showed decreased sperm count, sperm motility, sperm viability, and semen volume as well as altered sex hormones especially LH, FSH and testosterone (Figure 8). Previous studies demonstrated that ethanol induced toxic effects on male reproductive organs, sperm motility, volume and count (Akbari et al., 2017; Nishi et al., 2018; Tangsrisakda and Iamsaard, 2020). PA oil significantly ameliorated ethanol-induced altered levels of LH, FSH and testosterone, depicting its potential as a fertility-enhancing agent. This result is in accordance with the study of Rehman et al., who reported that PA extract consumption enhances testosterone production in males. Additionally sperm count, semen volume and sperm motility were also restored after PA oil consumption. Recently, Stoffel et al. reported that PUFAs, especially DHA and arachidonic acid are building blocks in the phospho- and sphingolipidomes of ovarian and testicular membranes for both female and male fertility (Stoffel et al., 2020). Moreover Collodel et al*.* identified that PUFAs like DHA, are positively correlated with sperm motility and count (Collodel et al., 2020).

Estradiol is the predominant and most biologically active estrogen, it modulates sexual behavior in the adult male, and it is also necessary for modulating libido, erectile function, and spermatogenesis (Schulster et al., 2016). We have found diminished levels of estradiol and increased prolactin levels in ethanol-treated group in-contrast to control. This observation is supported by the findings of Dosumu et al*.* who reported decreased reproductive hormone levels in male infertile rats treated with the ethanol (Dosumu et al., 2014). Male infertility model studies explained that ethanol successively produced male infertility by altering hormones and sperm motility (Akomolafe et al., 2017). While PA oil-treated group indicates improved hormone levels towards normal. This finding is in agreement with Adebayo et al. who reported that diabetic mice supplemented with almond presented increased testosterone level and sperm count possibly through improved cholesterol level (Adebayo et al., 2019).

Superoxide dismutase and glutathione have main roles to protect reproductive health and SOD is closely associated with oocyte maturation (Matos et al., 2009; Qureshi et al., 2019). The current study found raised SOD and glutathione levels after PA oil supplementation shown in Figure 8. Almonds might have favorable effects on inflammation because of the high content of PUFs and magnesium, which could regulate proinflammatory gene expression by diminishing prostaglandins, interleukins and ROS production (Kemse et al., 2014). 

RBCs and MCV levels were found to be decreased while WBCs and platelets counts were raised in ethanol-induced group as shown in Figure 7. These findings are in agreement with Osonuga et al. who explained that ethanol interferes with the production of white blood cells and reported to increase leukocyte count (Osonuga et al., 2010)*.* PA oil dosing significantly restored blood count towards normal in comparison to ethanol group. This finding is endorsed by another research that revealed that PA oil has an impact on the haemopoietic system due to the presence of PUFAs that believe to play an important role in blood cell production (Guo et al., 2004). Further, Limbkar et al*.* reported that PUFAs administration in mice stimulates the hematopoiesis and thrombopoiesis (Limbkar et al., 2017). Our results are in accordance with Koriem and El-Attar that discovered that almond consumption restored blood parameters especially WBCs (Koriem and El-Attar, 2022). 

Ethanol-treated animals have also shown increased LDL, TC and TG levels. Alcohol consumption causes disruption in lipid metabolism that increases LDL and cholesterol levels may be due to the obstruction in liver bile duct (Andreotti et al., 2008; Wang et al., 2010). Excessive alcohol intake shows marked disturbances in lipid metabolism that can lead to reduced semen count, sperm quality and motility (Finelli et al., 2021). In the present study, PA oil supplementation significantly reinstated the HDL levels, in contrast decreased cholesterol, TGs, LDL and VLDL levels as shown in Figure 7. Safarian et al. reported that almond husk significantly reduced total cholesterol and triglycerides that improve serum antioxidant capacity and sperm count (Safarian et al., 2016). 

The histological section of the current study showed degeneration of seminiferous tubules, germinal cells, and disorganized and irregular testicular membrane in ethanol-treated group. However, PA oil-treated animal testes showed improved architecture of germinal cell and seminiferous tubules (Figure 9). These findings are in accordance with Abd Elmeged and Mahzari*.* who reported almond consumption significantly reduced testicular damage and restored spermatogenesis by preventing the cadmium chloride-induced oxidative stress in rats (Abd Elmeged and Mahzari).

In conclusion, this study revealed that PA oil consumption had beneficial effects on both male and female fertility processes by augmenting ovulation and spermatogenesis with no toxic effect in both sexes. This could be associated to its PUFAs content that might improve hormones and antioxidant enzyme activities such as LH, FSH, SOD, and glutathione which improve infertility. Therefore, it is suggested that daily consumption of PA may provide an effective strategy to improve infertility. However, further studies are required to investigate the effects of its active constituents and pure compounds in clinical trials.

**Table 1:** Total phenolic content in *Prunus amygdalas*

Sample	Total Phenolic Content (mg/g GAE)
Prunus amygdalas	0.03

**Table 2:** Phytochemical analysis of *Prunus amygdalas*

Phytochemicals	P. amygdalas
Phlobatannins	+
Flavonoids	+
Glycosides	+
Alkaloids	-
Saponins	-
Phenols	+
Terpenoid	+
Tannin	-

The presence of phytochemicals is presented as +, while the absence is denoted as –

**Table 3:** GC-MS analytical results of *Prunus amygdalas* oil

S. No.	Name of compound	Retention time (min)	Peak area (%)	Molecular weight	Formula
1	3-Nonen-2-one	16.50	0.40	140	C9H160
2	2-Decenal (E)	17.03	0.54	154	C10H18O
3	2,4- Decadienal	19.12	2.60	152	C10H16O
4	Trans-3-Nonen-2-one	19.59	0.13	140	C9H16O
5	2-Decyn-1-ol	20.01	0.14	154	C10H18O
6	2,4,6-Trimethyloctane	20.39	0.49	156	C11H24
7	10-Undecenal	20.55	0.32	168	C11H20O
8	Cis-4,5-Epoxy-(E)-2-decenal	21.07	0.12	168	C10H16O2
9	Dodecanoic acid	27.15	0.33	200	C12H24O2
10	7-Hexadecene	30.02	0.07	224	C16H32
11	Palmitic acid, methyl ester	36.61	0.76	270	C17H34O2
12	Octadecanoic acid	38.27	8.81	284	C18H36O2
13	Conjugated linoleic acid	40.55	0.15	308	C20H36O2
14	7-Hexadecenoic acid	40.73	0.49	268	C17H32O2
15	9-Octadecenoic acid,1,2,3-propanetriyl ester	43.49	78.3	884	C57H104O6
16	Tricyclo triacontane, diepoxy	45.59	0.05	444	C30H52O2
17	Octadecenoic acid,2,3- dihydroxy propyl ester	52.85	2.45	356	C21H40O4
18	Clionasterol	57.46	1.49	414	C29H50O
19	5-androsten-17alpha-ethynyl-3 beta 17 beta-diol	57.88	0.09	314	C21H30O2
20	Alpha Tocopherol	59.99	0.12	430	C29H50O2
21	5-Cholenic acid-3 beta- ol	62.62	0.65	374	C24H38O3

**Table 4:** DPPH-radical scavenging potential of *Prunus amygdalas* oil at different concentrations: Ascorbic acid as positive control

Samples	Concentration (μg/ml)	% Inhibition (Mean±SD)
Prunus amygdalas Oil	10 100 200 300	58.10±2.85 50.33±6.05 32.95±3.68 22.36±2.19
Ascorbic acid (Standard)	10 100 200 300	76.16±0.93 81.91±1.57 85.59±0.55 88.24±1.29

**Table 5:** Percentage Mortality of *Prunus amygdalas *oil as compared to control and standard

Samples	Concentration	No. of Shrimps	No. of Survivors	% Mortality
Control (Distilled Water)	10	30	30	0
	100	30	30	0
	1000	30	30	0
Standard (Etoposide)	10	30	5	83.33
	100	30	1	96.6
	1000	30	0	100
Prunus amygdalas Oil	10	30	29	0
	100	30	26	3.33
	1000	30	25	6.66

**Table 6:** Effect of *Prunus amygdalas* on the number of pups/ litters of F_1_ and F_2_ generation in rats

F_1_				F_2_			
Control	T_1 _(1ml/kg)	T_2 _(2ml/kg)	p Value	Control	T_1_(1ml/kg)	T_2 _(2ml/kg)	p Value
4.33±0.61	5.17±0.47	5.83±0.30	p>0.05	4.33±0.21	6.17±0.30*	6.83±0.30*	p<0.05

n=6, Mean±SEM; *p<0.05 significant as compared to control. F_1_ presents Parent Generation Pups, while F_2_ presents 2^nd^ Generation pups; T_1_ shows low dose group whileT_2_ shows high dose group.

**Table 7:** Developmental findings in F_1_ and F_2 _generation Pups

Parameters	F_0_ Parents/F_1_ Pups			F_1_ Parents/F_2 _Pups			p-Value
	Control	T1 (1ml/kg)	T2 (2ml/kg)	Control	T1 (1ml/kg)	T2 (2ml/kg)	
No. of Pregnant Rats	5	6	6	6	6	6	p> 0.05
^a ^Litter Size	5.4	4.16	4.83	4.16	5.33 *	6 *	p< 0.05
^b^ Fertility Index (%)	83.3	100	100	100	100	100	p> 0.05
^c^ Live Birth Index (%)	100	100	96.6	100	100	100	p> 0.05
^d^ Survival index at day 4 (%)	100	100	100	100	100	100	p> 0.05
^e^ Survival index at day 21 (%)	100	100	100	100	100	100	p> 0.05

F_0 _presents Parent Generation, while F_1_ presents 1^st^ Generation Pups, while F_2_ presents 2^nd^ Generation Pups, T_1_ shows low dose group while T_2_ shows high dose group. n = 6, Mean±SEM; *p<0.05 significant, as compared to control. ^a^ Litter size = count of pups/count of gestated females, ^b^ Fertility index (%) = (Females count giving birth/females count lived together) × 100, ^c^ Live birth index (%) = (count of pups alive at day 0/count of pups born) × 100, ^d^ Survival index - 4 days (%) = (count of pups alive on day 4/ day 0 alive pups count) × 100,

^ e^ Survival index- 21 days (%) = (count of alive pups on day 21/ day 4 alive pups count) × 100

**Table 8:** Male fertility parameters

Parameters	Control	Ethanol (2 g/Kg/d)	T_1 _(Vit E-105 IU/d)	T_2 _(Prunus amygdalas- (2 ml /Kg/d)	p value
Semen pH	7.23±0.02	7.25±0.03	7.54±0.13	7.27±0.29	p>0.05
Semen Volume (ml)	2.06±0.02	1.28±0.05	2.35±0.04	2.51±0.06 *	p<0.05
Sperm Count (×10^6^ sperm/ml)	62.1±1.24	32.4±5.84	61.49±6.69 *	89.5±8.32 **	p<0.01
Sperm viability (%)	60.6±5.60	23.4±2.76	67.4±6.15 *	95.8±6.96 **	p<0.01
Sperm Motility (%)	50.90±3.22	30.31±3.72	62.41±5.32 *	82.32±6.81 **	p<0.01

n=6, Mean±SEM; *p<0.05 and **p<0.01 significantly different from control group.
